# Maternal Protein Restriction and Branched-Chain Amino Acid Supplementation Differentially Affect Maternal Energy Balance and Impair Offspring Growth

**DOI:** 10.3390/nu18020322

**Published:** 2026-01-20

**Authors:** Daniela Redrovan, Souvik Patra, Md Tareq Aziz, Matthew W. Gorton, Emily A. Chavez, Scott Frederiksen, Joshua Rowe, Adel Pezeshki, Prasanth K. Chelikani

**Affiliations:** 1 School of Veterinary Medicine, Texas Tech University, Amarillo, TX 79106, USA; dredrova@ttu.edu (D.R.); soupatra@ttu.edu (S.P.); mdtaziz@ttu.edu (M.T.A.); emily.a.chavez@ttu.edu (E.A.C.); scott.frederiksen@ttu.edu (S.F.); joshrowe@ttu.edu (J.R.); 2 Department of Animal and Food Sciences, Oklahoma State University, Stillwater, OK 74078, USA; matthew.gorton@okstate.edu (M.W.G.); adel.pezeshki@okstate.edu (A.P.)

**Keywords:** protein restriction, branched-chain amino acids, pregnancy and lactation, craniofacial growth, low birth weight

## Abstract

Background: The increasing prevalence of low-birth-weight (LBW) offspring from obese mothers underscores the need for dietary strategies to mitigate the transgenerational propagation of metabolic diseases. Objectives: We determined whether dietary protein restriction under obesogenic conditions altered maternal energy balance and led to LBW offspring and whether branched-chain amino acid (BCAA) supplementation improved maternal energy balance and mitigated weight and craniofacial skeletal deficits in offspring. Methods: High-fat-fed obese pregnant Sprague Dawley rats (~8–10 weeks of age, *n* = 8–11/group) were randomized in study 1 to control high-fat diet (20% protein; HFD), low-protein diet (LP; 5% protein), and LP + BCAA diet (100% BCAA requirements) and in study 2 to control HFD (20% protein), LP (10% protein), and LP + 2BCAA diet (200% BCAA requirements). Post-weaning offspring were fed HFD until 8 weeks of age. Results: Protein restriction promoted hyperphagia and energy expenditure, whereas BCAA supplementation attenuated such hyperphagic effects in pregnancy but not in lactation. Protein restriction reduced maternal body weight in lactation, and although BCAA supplementation did not reverse the weight loss, it enhanced insulin sensitivity and paradoxically reduced offspring survival. Maternal protein restriction reduced offspring body weight and craniofacial bone growth that persisted into adulthood, but BCAA supplementation did not rescue such deficits. Conclusions: Maternal protein restriction in obese dams enhanced maternal energy expenditure but impaired offspring growth and development. Although BCAA supplementation improved maternal energy balance, it was insufficient to reverse the adverse effects of maternal protein restriction on offspring growth under obesogenic conditions.

## 1. Introduction

Maternal nutrition is important for a healthy pregnancy, for promoting ideal fetal growth, and for improving the long-term health of both the mother and the child [1]. In the United States, the prevalence of pre-pregnancy obesity increased by 11% from 2016 to 2019 [2], and 58% of all delivering mothers were obese in 2021 [3]. On the contrary, inadequate or poor maternal nutrition is associated with abnormal fetal growth patterns, such as low birth weight (LBW, <2500 g) [1]. Other determinants of LBW in low-income countries include rural residence, delayed initiation of antenatal care, pregnancy-induced hypertension, insufficient iron and folic acid supplementation, and poor dietary diversity [4]. Though LBW is often associated with chronic maternal undernutrition, the rates of LBW were 8.16% in women with class I obesity, 8.68% in women with class II obesity, and 9.67% in women with class III obesity, indicating that maternal overnutrition can also lead to LBW [5,6,7]. Thus, there is a need for dietary strategies that can reverse the detrimental effects of maternal obesity on the metabolic health of offspring in adulthood.

Maternal protein restriction and nutritional deficiencies during pregnancy and lactation have profound effects on both maternal and offspring metabolic health. For example, maternal low protein diets increased maternal food intake [8], and in offspring reduced birthweight and increased food intake [9,10,11,12] and likely energy expenditure at adulthood in rats [13], and impaired glucose tolerance, decreased insulin sensitivity, and reduced serum cholesterol levels in mice at weaning [14]. However, whether maternal protein restriction alters both maternal and offspring energy expenditure and substrate utilization is largely unknown. Moreover, fetal adaptations to low-protein environments when followed by rapid postnatal catch-up growth in mouse offspring result in long-term consequences, including obesity and type 2 diabetes [15]. Offspring bone development is also compromised, as maternal protein restriction has been shown to increase dentin thickness, reduce dental pulp vascularity and alveolar bone area, and alter the expression of bone regulatory molecules, indicating a systemic phenotype that limits both organ and skeletal growth [16]. Although the effects of maternal protein restriction on offspring growth have been well-documented, whether maternal dietary alterations can mitigate such adverse programming effects on offspring has received little attention.

Currently, the strategies used to optimize the growth and development of LBW infants include the provision of breast milk and donor milk, milk fortification, kangaroo mother care, probiotic and micronutrient supplementation, and enteral feeding to meet protein and energy targets [17,18,19]. Despite existing protein recommendations, optimal intake levels have not been fully defined, and important gaps remain regarding the composition of protein and the optimal strategies for delivering nutrients in the postnatal period in humans [18,20]. In a systematic review on the effects of nutritional interventions during the preconception and pregnancy period, only supplementation with iron, folic acid, and whole foods appeared to favor the intervention in reducing LBW, though with very low certainty [21]. In search of more effective strategies to mitigate LBW, various dietary interventions have been explored in animal models. For example, the neurobehavioral and cognitive deficits in offspring from protein-restricted mothers appear to be attenuated by maternal supplementation of choline in mice [22] and by maternal gavage of Spirulina algae in rats [23]. Dietary folic acid has been shown to attenuate the hepatic transcriptional changes in offspring born to protein-restricted rat dams [24]. However, the effects of these interventions on the maternal and offspring energy balance remain poorly understood.

Dietary protein supplementation to energy- [25] or protein-restricted [26] rat dams has been shown to positively influence fetal and postnatal growth metrics, organ mass, and metabolic indicators in offspring. To further define the dietary protein components that confer growth and metabolic benefits to LBW offspring, the effects of individual amino acid components of dietary protein have been studied in several animal models. For example, supplementation of taurine (2.5% wt/v) in water to protein-restricted rat dams has been shown to improve β-cell function in male offspring and insulin sensitivity in female offspring [27], prevent loss of pancreatic islets and morphological deterioration of mitochondria in the β-cell in offspring [28], and protect against high blood pressure in male offspring [29]. Similarly, supplementation of glycine (3% wt/wt), but not alanine, was shown to protect against an increase in BP in offspring of protein-restricted dams [30], and dietary L-citrulline (2 g/kg/wt/d) increased fetal growth and muscle protein synthesis, whereas L-arginine or the non-essential amino acids (alanine, histidine, glycine, and serine) were ineffective in altering fetal growth in rats [31]. Elevated plasma branched-chain amino acid (BCAA) (leucine, isoleucine, and valine) levels have been associated with various metabolic disorders, including obesity, insulin resistance, type 2 diabetes mellitus, and gestational diabetes mellitus [32]. Serum levels of leucine, isoleucine, and valine were reported to be higher in the first trimester of pregnancy in women with gestational diabetes mellitus [33]. In contrast, BCAA levels were reduced in the circulation of protein-restricted mouse dams [34]. The effects of dietary BCAA on maternal and offspring metabolic health under protein-restricted conditions are inconsistent. For example, dietary supplementation of BCAA (2% wt/wt) to protein-restricted mice increased fat mass and liver protein content in dams but did not rescue LBW and reduced liver protein content in the offspring [35]. In contrast, dietary BCAA (1–1.8% wt/wt) to protein-restricted rat dams partially reversed the reduced body weight, fat mass, and organ weights in the pups [36]. In studies to date, the effects of BCAA and other interventions to rescue LBW offspring were examined under conditions when the dams and offspring were fed normocaloric diets, which model human conditions of dietary caloric and protein insufficiency. Though the incidence of macrosomia, or being large for gestational age, is greater with obese pregnancies [37], 8–10% of the offspring born to obese mothers are also LBW [5,6,7], and the current interventions do not model this maternal obesogenic environment that leads to LBW offspring.

It is largely unknown whether dietary supplementation of BCAA to protein-restricted obese dams alters caloric intake and energy expenditure during pregnancy and lactation and affects offspring growth. We hypothesized that maternal dietary protein restriction under obesogenic conditions impairs offspring growth and that supplementation with BCAA will mitigate the effects of protein restriction on maternal energy balance and reverse the adverse effects of maternal obesity on offspring growth and skeletal development. Our objective was to determine the effects of a low-protein diet and BCAA supplementation to obese dams on maternal energy balance (caloric intake, energy expenditure, and respiratory quotient), body weight, glucose and insulin tolerance, pregnancy rate and litter size, as well as offspring body weight and craniofacial bone development.

## 2. Materials and Methods

### 2.1. Animals, Housing, and Diets

**Figure 1:**
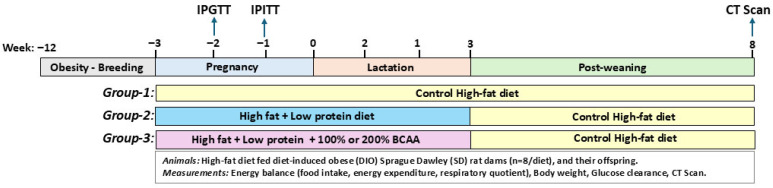
Experimental design. Following acclimatization to housing conditions, 3-week-old female Sprague Dawley (SD) rats were fed a control high-fat diet (40% fat kcal; HFD) for 8 weeks to induce obesity and bred with chow-fed males. In study 1, pregnant diet-induced obese (DIO) SD rats were randomized to three HFD groups: (1) control HFD (20% protein; *n* = 8), (2) low-protein (5% protein, LP; *n* = 11), and (3) LP + BCAA (LP + 100% requirement for branched-chain amino acids; *n* = 11). In study 2, a separate cohort of pregnant DIO SD rats were randomized to three HFD groups: (1) control HFD (20% protein; *n* = 8), (2) low-protein (10% protein, LP; *n* = 8), and (3) LP + 2BCAA (LP + 200% requirement for branched-chain amino acids; *n* = 8) during pregnancy and lactation. Offspring received the control HFD post-weaning for 8 weeks. Measurements included energy balance (food intake, energy expenditure, and respiratory quotient of dams in the first week after breeding and for ~5 days before and after the expected date of parturition), daily food intake and weekly body weight of dams and offspring, intraperitoneal glucose tolerance test (IPGTT) in week 2 of pregnancy, intraperitoneal insulin tolerance test (IPITT) in week 3 of pregnancy in dams, and CT scans of skull in offspring at 8 weeks of age.

Experimental procedures on animals were approved by the Texas Tech University Health Science Center Institutional Animal Care and Use Committee (IACUC protocol #21022), and the ARRIVE reporting guidelines were followed [38]. Female Sprague Dawley (SD) rats (3 weeks old, Charles River, Wilmington, MA, USA) were housed in pairs in shoebox microisolator cages under controlled environmental conditions (temperature 22–25 °C, humidity 21–24%, 12 h light–dark cycle with lights off at 1230 h) and fed with a high-fat diet (40% fat kcal; control) from 3 to 8 weeks of age to develop diet-induced obese (DIO) rats. Daily animal care and maintenance were conducted between 1030 and 1230 h, and food and water were provided *ad libitum* throughout the study. Females were paired 1:1 with age-matched chow-fed males and co-housed for 5 days. In study 1, pregnant DIO SD rats were randomized to three high-fat diet (40% fat kcal; HFD) groups (Table 1): (1) control HFD (20% protein; *n* = 8), (2) low-protein (5% protein, LP; *n* = 11), and (3) LP + BCAA (LP + 100% requirement for branched-chain amino acids; *n* = 11). Based on the outcomes observed with 100% BCAA, we reasoned that the amount of BCAA provided was likely insufficient to alter the offspring phenotype, and hence, in the next study, we included an experimental group receiving a higher 200% BCAA dose. Therefore, in study 2, using a similar design, a separate cohort of pregnant DIO SD rats were randomized to three HFD groups (Table 1): (1) control HFD (20% protein; *n* = 8), (2) low-protein (10% protein, LP; *n* = 8), and (3) LP + 2BCAA (LP + 200% requirement for branched-chain amino acids; *n* = 8) during pregnancy and lactation. Offspring received the control HFD post-weaning till 8 weeks of age (Figure 1). All diets were prepared in-house according to AIN-93 guidelines [39] and stored at 4 °C until further use. Regarding sample size, based on data from our previous studies [40,41,42], in a randomized design with α = 0.05, the effect size, SD, power, and sample size for body weight were 0.93, 15.53 g, 99%, and 8 rats. For body fat, they were 1.49, 4.37 g, 100%, and 6 rats, as generated by Systat11^®^ (Systat Software Inc., Palo Alto, CA, USA). The authors were not blinded to the group allocation during animal experimentation and data analyses.

### 2.2. Food Intake, Respiratory Quotient, and Energy Expenditure

Energy expenditure (EE) and respiratory quotient (RQ) were measured using the CLAMS^®^ system (Columbus Instruments, Columbus, OH, USA) by indirect calorimetry. Oxygen consumption (VO_2_; mL/kg body weight/h) and carbon dioxide production (VCO_2_; mL/kg body weight/h) were recorded for each dam from 1230 h on the previous day to 1030 h the following day (Figure 1). Rats were housed individually in CLAMS^®^ for the first week after breeding and for ~5 days before and after the expected date of parturition (average gestation length: 21 days). The CLAMS^®^ system parameters were set to a 2 L/min sample flow, cage measure every 30 sec, and sampling interval of 82–142 min, yielding 14–16 readings per day. Total EE was calculated using the following equation: EE (kcal/h) = (3.815 × VO_2_ (L/h)) + (1.232 × VCO_2_ (L/h)) as described previously [43]. Daily food intake was measured manually between 1030 h and 1230 h using a conventional scale (Ohaus Corporation, model CL 5000, Parsippany, NJ, USA).

### 2.3. Body Weight, Intraperitoneal Glucose, and Insulin Tolerance Tests

Body weight of mothers and pups was recorded weekly between 10:30 and 12:30 h using a conventional scale (Ohaus, model CL 5000). The intraperitoneal glucose tolerance test (IPGTT) was performed in the second week of pregnancy. Briefly, after a 12 h fast, rats received an intraperitoneal (IP) injection of a 50% dextrose solution at 2 g/kg, followed by blood glucose measurements from a tail snip using a handheld glucometer (ReliOn™ Premier Classic, Blood Glucose Test Strips) at 0 (baseline), 15, 30, 60, and 120 min post-injection. The IP insulin tolerance test (IPITT) was performed in the third week of pregnancy. After a 6 h fast, rats received IP human insulin (Sigma-Aldrich, Burlington, MA, USA; 0.75 IU/kg), followed by blood glucose measurements from a tail snip using a handheld glucometer every 10 min for 1 h post-injection (Figure 1).

### 2.4. Computed Tomography Scan

Craniofacial measurements of pups at 8 weeks of age were obtained using high-resolution helical computed tomography (CT) scans (Toshiba Aquilion TSX-101A) and a commercially available medical imaging software platform (Osirix MD, UDI-PI: 14.1.2, Paris, France). Scanswere performed in sternal recumbency at a voltage of 100 kV, current of 200 mA, exposure ime of 750 msec, pitch of 0.656, and slice thickness of 0.5 mm. Measurements included overall skull length, facial and neurocranial lengths (rostral and caudal to the nasion, respectively), overall skull width (at the widest point of the zygomatic arches), facial width (at the level of the infraorbital fissure), and neurocranial width (immediately caudal to the zygomatic process of the temporal bone).

### 2.5. Statistical Analyses

Data, including food intake, energy expenditure, body weight, IPGTT, IPITT, and skull measurements, were analyzed using linear mixed models with fixed effects of diet, treatment, time, treatment × interactions, and repeated measurements on time (or space for skull measurements), which were modeled as a first-order autoregressive covariance structure in SPSS (IBM SPSS Statistics, version 24.0, Chicago, IL, USA). Energy expenditure was analyzed with body weight as a covariate. The Grubbs’ test was used to identify and exclude outliers for the various energy balance and metabolic readouts (GraphPad Prism 10.1, San Diego, CA, USA). ANOVA or repeated-measures mixed models with post hoc comparisons were conducted using the Benjamini–Hochberg procedure, with significance set at α = 0.05 (GraphPad Prism version 10.01). Chi-square tests were used to analyze pup survival and pregnancy success, while Fisher’s exact test was used to assess group differences for the number of pups born alive or dead and the incidence of cannibalism (GraphPad Prism version 10.01). Differences were considered significant at *p* ≤ 0.05, and trends at *p* ≤ 0.10.

## 3. Results

### 3.1. Maternal Protein Restriction and Branch Amino Acid Supplementation Alter Food Intake, Energy Expenditure, and Respiratory Quotient During Pregnancy and Lactation

**Figure 2:**
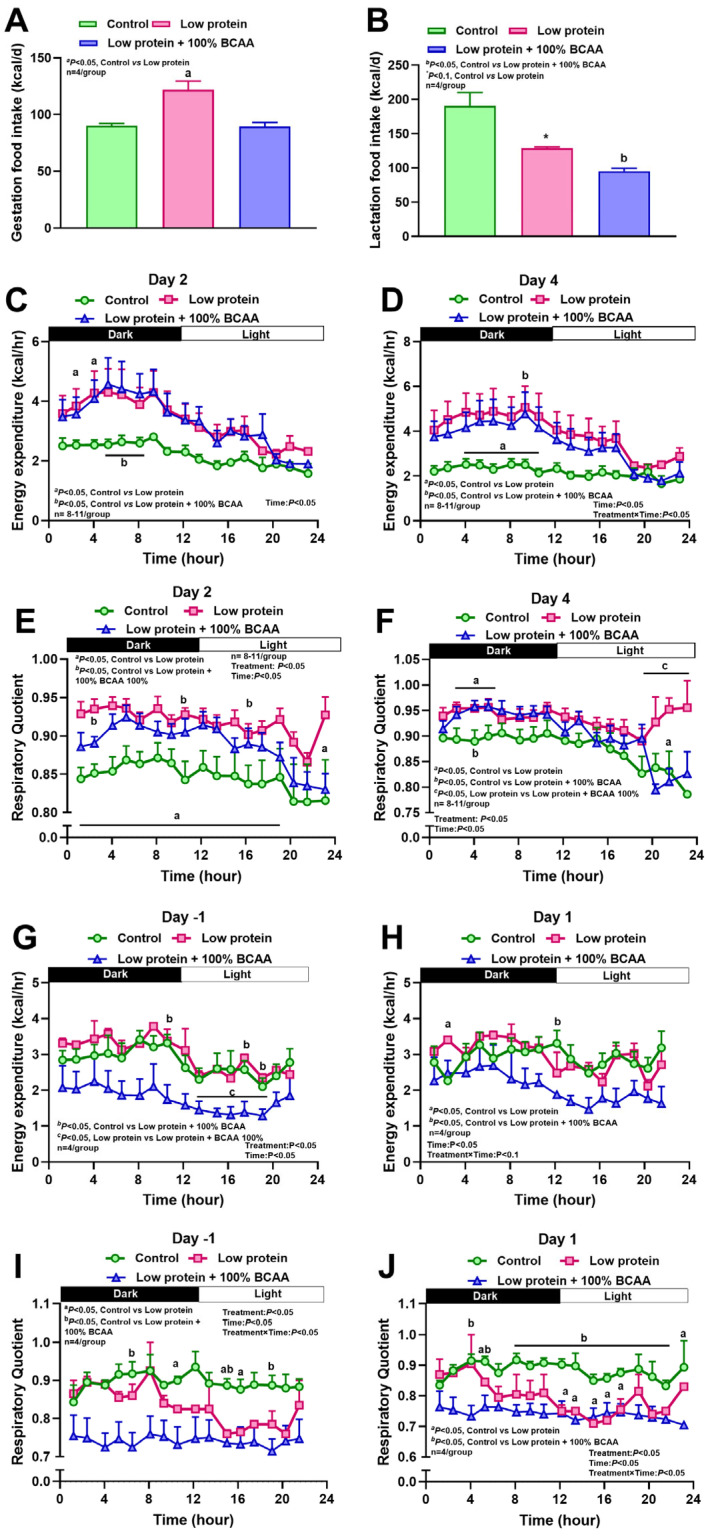
Effects of maternal dietary interventions in study 1 on (**A**) average daily food intake in gestation, (**B**) average daily food intake in lactation, (**C**) representative energy expenditure on day 2 of gestation, (**D**) representative energy expenditure on day 4 of gestation, (**E**) representative respiratory quotient on day 2 of gestation, (**F**) representative respiratory quotient on day 4 of gestation, (**G**) energy expenditure on day 1 prior to parturition, (**H**) energy expenditure on day 1 of lactation, (**I**) respiratory quotient on day 1 prior to parturition, and (**J**) respiratory quotient on day 1 of lactation. Pregnant diet-induced obese (DIO) SD rats were fed control HFD (*n* = 8), low-protein diet (5% protein, *n* = 11), and LP + BCAA (LP + 100% requirement for branched-chain amino acids, *n* = 11). ^a,b^ *p* < 0.05 vs. control, ^c^ *p* < 0.05 low-protein vs. LP + BCAA, * *p* < 0.10 vs. control. Values are mean ± SEM, n = 8–11.

In study 1, average caloric intake during pregnancy increased by 35% in LP dams compared to control dams, whereas LP + BCAA dams did not differ from controls (Figure 2A). During lactation, intake tended (*p* < 0.1) to be reduced by 32% in LP dams and significantly (*p* < 0.05) reduced by 49% in LP + BCAA dams compared to control (Figure 2B). Energy expenditure (EE) increased in LP and LP + BCAA rats compared to controls from the second day of diet intervention (Figure 2C,D, Supplemental Figure S1A–E). Similarly, from the first day, LP and LP + BCAA rats had higher respiratory quotient (RQ) values compared to controls, suggesting a shift in substrate use towards carbohydrate utilization. Interestingly, LP + BCAA rats had decreased RQ during the light period, suggesting a reversal in energy substrate utilization to lipids in lactation (Figure 2E,F, Supplemental Figure S1F–J). At birth (day 1 of lactation), control and LP dams did not differ in EE; LP + BCAA dams had reduced EE compared to both groups, and LP and LP + BCAA dams had lower RQ values suggestive of a substrate shift to lipid use (Figure 2G–J, Supplemental Figure S2A–F).

**Figure 3:**
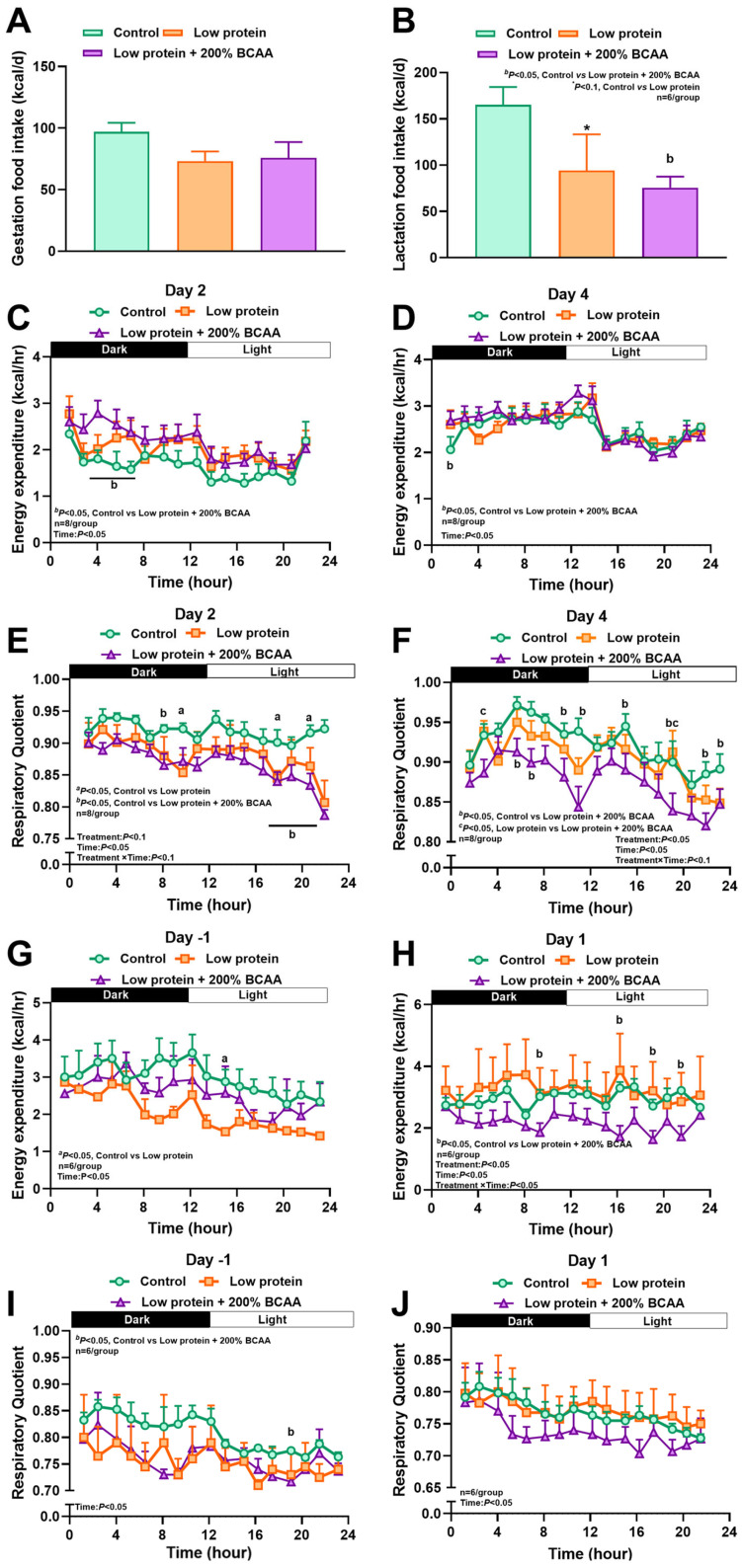
Effects of maternal dietary interventions in study 2 on (**A**) average daily food intake in gestation, (**B**) average daily food intake in lactation, (**C**) representative energy expenditure on day 2 of gestation, (**D**) representative energy expenditure on day 4 of gestation, (**E**) representative respiratory quotient on day 2 of gestation, (**F**) representative respiratory quotient on day 4 of gestation, (**G**) energy expenditure on day - 1 prior to parturition, (**H**) energy expenditure on day 1 of lactation, (**I**) respiratory quotient on day -1 prior to parturition, and (**J**) respiratory quotient on day 1 of lactation. Pregnant diet-induced obese (DIO) SD rats were fed control HFD (20% protein, *n* = 8), low-protein diet (10% protein, LP; *n* = 8), and LP + 2BCAA (LP + 200% requirement for branched-chain amino acids; *n* = 8) during pregnancy and lactation. ^a,b^ *p* < 0.05 vs. control, ^c^ *p* < 0.05 low-protein vs. LP + BCAA, * *p* < 0.10 vs. control. Values are mean ± SEM, *n* = 8.

In study 2, average daily caloric intake during pregnancy did not differ among groups, and similar to study 1, intake during lactation tended (*p* < 0.1) to be reduced by 42% in LP dams and by 54% in LP + 2BCAA dams (*p* < 0.05) (Figure 3A,B). Compared to controls, LP + 2BCAA rats had a brief increase in EE during the dark period on the second and fourth days of diet intervention and decreased RQ during the first three days in the light period (Figure 3C–F, Supplemental Figure S3A–J). At birth, LP dams had a brief reduction in EE during the light period, whereas LP + 2BCAA dams decreased EE on the second day and reduced RQ compared to controls (Figure 3G–J, Supplemental Figure S4A–F). These findings indicate that dietary protein restriction in study 1 promoted hyperphagia, and BCAA supplementation partly attenuated such hyperphagic effects. Further, protein restriction in both studies increased energy expenditure and shifted substrate use toward carbohydrate in pregnancy, whereas, in early lactation, food intake and energy expenditure were decreased by protein restriction and BCAA supplementation.

### 3.2. Maternal Protein Restriction and BCAA Supplementation Reduced Body Weight, and BCAA Supplementation Increased Insulin Sensitivity

**Figure 4:**
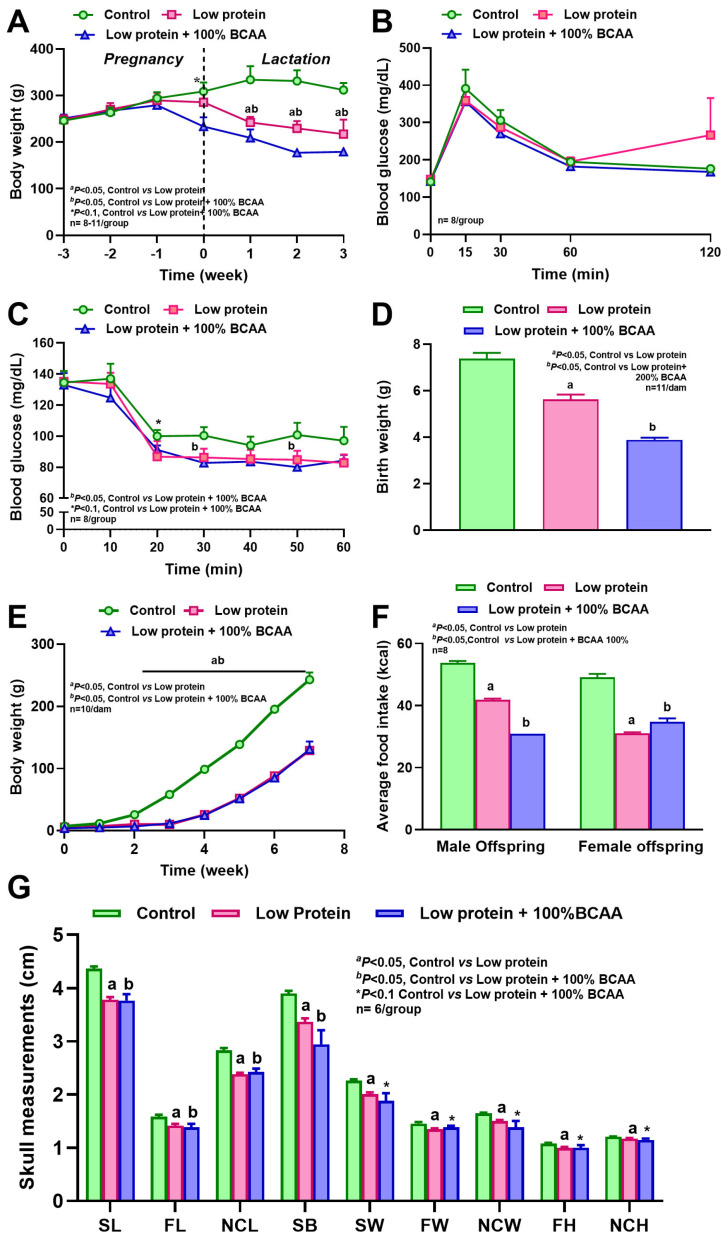
Effects of maternal dietary interventions in study 1 on (**A**) body weight of dams during pregnancy and lactation, (**B**) blood glucose concentrations after intraperitoneal glucose tolerance test in week 2 of gestation, (**C**) blood glucose concentrations after intraperitoneal insulin tolerance test in week 3 of gestation, (**D**) birth weight of offspring, (**E**) body weight of offspring till 8 weeks of age, (**F**) average food intake of male and female offspring, and (**G**) skull measurements of offspring at 8 weeks of age. Craniofacial measurements in the CT scan included skull length (SL), facial length (FL), neurocranial length (NCL), skull base (SB), skull width (SW), facial width (FW), neurocranial width (NCW), facial height (FH), and neurocranial height (NCH). Pregnant diet-induced obese (DIO) SD rats were fed control HFD (*n* = 8), low-protein diet (5% protein, *n* = 11), and LP + BCAA (LP + 100% requirement for branched-chain amino acids, *n* = 11). ^a,b^ *p* < 0.05 vs. control, * *p* < 0.10 vs. control. Values are mean ± SEM.

**Figure 5:**
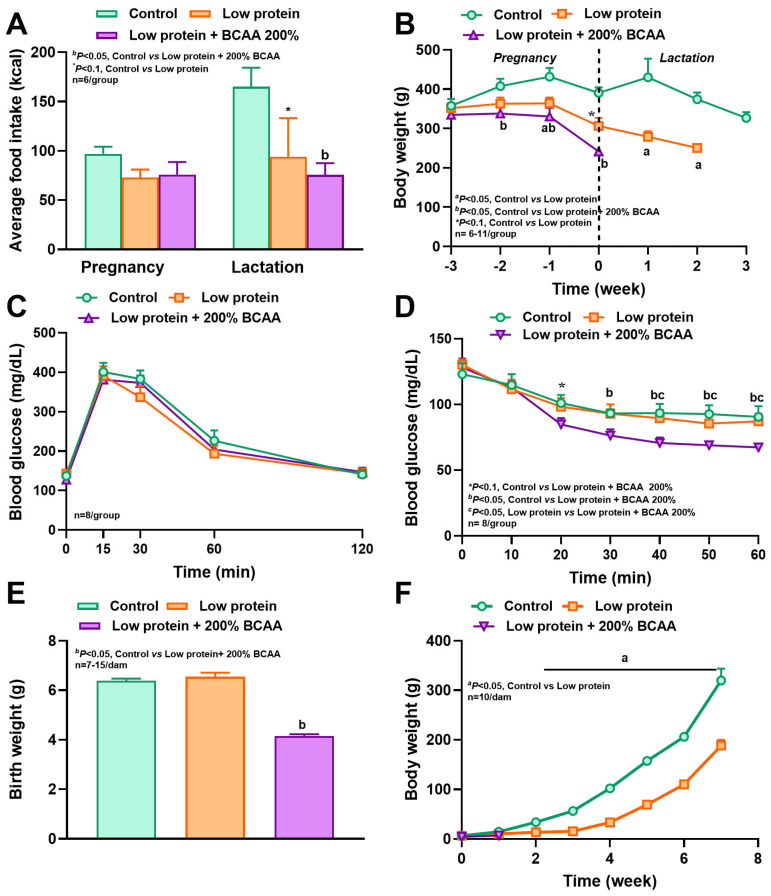
Effects of maternal dietary interventions in study 2 on (**A**) average food intake of dams in pregnancy and lactation, (**B**) body weight of dams during pregnancy and lactation, (**C**) blood glucose concentrations after intraperitoneal glucose tolerance test in week 2 of gestation, (**D**) blood glucose concentrations after intraperitoneal insulin tolerance test in week 3 of gestation, (**E**) birth weight of offspring, and (**F**) body weight of offspring. ^a,b^ *p* < 0.05 vs. control, ^c^ *p* < 0.05 low-protein vs. LP + BCAA, * *p* < 0.10 vs. control. Values are mean ± SEM, *n* = 8.

In study 1, during the first week of lactation, we observed a weight loss of 28% in the LP dams and 37% in the LP + BCAA dams compared to the control, but body weights did not differ among groups during pregnancy (Figure 4A). In study 2, the weights of LP + 2BCAA dams were decreased by 16% in the first week of pregnancy and 41% in the first week of lactation, and the weights of LP dams were reduced by 15% in the second week of pregnancy and 34% during lactation, with a concurrent reduction in food intake, compared to the control (Figure 5A,B). Though the blood glucose excursions following the IPGTT did not differ among groups (Figure 4B and Figure 5C), the IPITT revealed that the LP + BCAA group had 18–21% lower blood glucose at 20, 30, and 50 min compared to the control (Figure 4C). Similarly, the IPITT study revealed that the LP + 2BCAA had 18–26% lower blood glucose compared to the control and 21–23% lower glucose compared to LP (Figure 5D). As the LP + 2BCAA mothers cannibalized their offspring in the first week of lactation (Figure 5F), they were not included thereafter. These findings show that maternal protein restriction and BCAA supplementation reduced body weight during lactation, but notably, BCAA supplementation enhanced insulin sensitivity in the dams.

### 3.3. Maternal BCAA Supplementation to Low-Protein Diets Reduced Litter Size and Offspring Survival

In studies 1 and 2, the pregnancy rates were similar among groups (Table 2). Litter size was reduced in LP by 49% in study 2 (Table 2) and tended (*p* < 0.1) to be reduced by 18–24% in LP + BCAA and LP + 2BCAA compared to the respective control groups. The proportion of pups born alive was lower with 87% in LP + 2BCAA compared to the 100% control group (Table 2), and conversely, pups’ survival at weaning was severely impaired, as 0% survived in LP + 2BCAA compared to 89% survival in the control group from birth till weaning. The LP + 2BCAA group had a greater incidence of cannibalism than other groups. These results indicate that BCAA supplementation to low-protein diets reduces litter size and offspring viability.

### 3.4. Maternal Protein Restriction Affected Offspring Body Weight and Reduced Craniofacial Bone Dimensions

**Figure 6:**
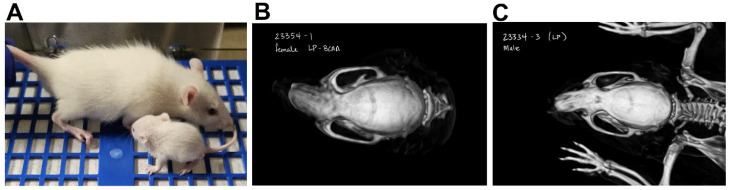
Effects of maternal dietary interventions on representative offspring. (**A**) Size difference between control and low-protein pups at weaning (control, top; low-protein diet, bottom). (**B**) Nasomaxillary deviation in LP + BCAA pup at 8 weeks of age. (**C**) Nasomaxillary deviation in LP pup at 8 weeks of age.

The average caloric intakes of male and female offspring from LP dams decreased by 20–38%, and intakes of males and females from LP + BCAA dams decreased by 29–42%, compared to controls (Figure 4F). The birth weight of LP pups reduced by 24%, LP + BCAA pups by 47%, and LP + 2BCAA pups by 44%, compared to the control (Figure 4D and Figure 5E). This difference in body weight persisted, as the LP and LP + BCAA pups continued to have lower weights compared to the control group until 8 weeks of age (Figure 4E, Figure 5F, and Figure 6A). CT scans of craniofacial measurements revealed differences in bone growth in certain areas (Figure 4G). The length of the skull, face, neurocranium, and skull base was 14–16% shorter in the LP and LP + BCAA groups compared to the control group. The width of the skull, face, and neurocranium, and facial and neurocranial height, were shorter in the LP group, whereas the LP + BCAA group showed only a tendency (*p* < 0.1) toward reduced bone dimensions compared to the control group. Furthermore, the offspring were growth-stunted, and there were nasomaxillary deviations found in the pups from protein-restricted dams (Figure 6, Supplemental Figure S5A,B). These findings demonstrate that maternal protein restriction reduced offspring body weight and craniofacial bone dimensions, with effects persisting beyond birth into postnatal development.

## 4. Discussion

Maternal programming of low birth weight with dietary protein restriction has been well documented in rodent models [14,15,44]; however, little is known about maternal adaptations in energy balance under obesogenic conditions and whether BCAA supplementation can rescue the offspring from adverse maternal impact. We demonstrate that maternal protein restriction, with or without BCAA supplementation, profoundly alters maternal energy balance and offspring development. First, we found that dietary protein restriction promoted hyperphagia and energy expenditure, and BCAA supplementation partly attenuated such hyperphagic effects in pregnancy. In contrast, during lactation, protein restriction and BCAA supplementation suppressed food intake and energy expenditure. Second, protein restriction reduced maternal body weight during lactation. Although BCAA supplementation did not attenuate the weight loss from protein restriction, it enhanced insulin sensitivity in dams but was paradoxically associated with reduced litter size and offspring viability and increased offspring cannibalization. Third, maternal protein restriction impaired offspring growth, with deficits persisting in postnatal life, including reduced craniofacial bone dimensions. Together, these results highlight that maternal protein restriction improved maternal energy balance but impaired reproductive success, offspring growth, and craniofacial skeletal development, and BCAA supplementation was ineffective in rescuing these metabolic and craniofacial deficits in offspring but enhanced maternal insulin sensitivity.

Previous studies have shown that dietary protein restriction in Sprague Dawley rat dams during pregnancy and lactation resulted in a slight increase in food intake during gestation [8,45] but a marked decrease in food intake through lactation, likely due to the high energetic demands of milk production [8,46]. Consistent with these studies, we also found that maternal protein restriction increased food intake in dams during gestation, especially in study 1, and decreased intake in lactation. In study 2, the inclusion of alanine in the low-protein diet to make it isonitrogenous to BCAA likely explains the lack of hyperphagic response to dietary protein restriction in gestation. Nonetheless, the hyperphagic response to protein restriction during gestation in study 1 and the attenuation of such effects by BCAA together support the protein leverage hypothesis that has been frequently observed in males [41,42,47]. Interestingly, BCAA supplementation further exacerbated the hypophagia in lactation, suggesting that BCAA was unable to rescue the adverse effects of protein restriction on lactational energy loss with a consequent limitation of caloric intake.

The homeorhetic adaptations in energy expenditure (EE) during the transition from pregnancy to lactation remain poorly understood. For example, pregnant hamsters were reported to have greater EE in the final third of gestation, along with a reduction in respiratory quotient (RQ), suggesting a shift towards lipid use at and after parturition [48]. Maternal dietary protein restriction was reported to decrease EE in lactating dams [46] and increase EE in offspring at adulthood [49]; however, in the former study, EE was estimated from carcass composition, and in the latter report, EE was predicted to increase based on the observed weight loss. We are not aware of any reports of direct measures of EE and RQ under conditions of maternal protein restriction. For the first time, we demonstrate that maternal protein restriction increased EE and promoted a shift toward carbohydrate utilization in pregnancy, whereas 2BCAA supplementation promoted a shift toward fat utilization and reduced EE at parturition. Our findings of increased EE with dietary protein insufficiency are, in general, consistent with previous studies in males [41,42,47]; however, the lack of an effect of alanine in study 2 and BCAA in both studies in attenuating such responses suggests that alanine and BCAA are unlikely to mediate the thermogenic effects of dietary protein restriction in pregnancy. The reduction in EE and RQ with protein restriction and BCAA in lactation is suggestive of a defense of a reduced body weight in the face of decreased caloric intake and increased lactational demands. In support of this, we found that both protein restriction and BCAA supplementation resulted in maternal weight loss during lactation, which is consistent with previous studies on weight loss in dams from dietary protein restriction [8,50].

There is substantial evidence that obesity, diabetes, and related metabolic diseases are associated with increased circulating BCAA [51]. Most of these studies were on non-pregnant subjects, and less is known about the role of BCAA in pregnancies with metabolic diseases. Circulating BCAAs were reported to be decreased from early to late pregnancy, with either no change or a concurrent increase in insulin resistance, in healthy women [52] and women with obesity [53]. In contrast, in women with gestational diabetes, circulating BCAA levels were reported to be increased in early pregnancy but not in later stages in some studies [32,33] but not others [54]. We observed an improvement in glucose clearance following insulin tolerance tests in pregnant dams supplemented with BCAA, with the effect being more pronounced with the inclusion of BCAA at 200% of the requirements (2BCAA). However, as 2BCAA decreased the body weight of dams, whether the improved glucose clearance is secondary to weight loss cannot be discerned from our study. Nonetheless, our findings support the notion that supplemental BCAA improved insulin sensitivity under conditions of protein restriction and obesogenic diets during the prepartum period. Moreover, the reproductive performance was adversely affected, with protein restriction and BCAA supplementation reducing litter size and offspring viability, and 2BCAA further exacerbating these deficits and concurrently increasing cannibalism. Dietary protein restriction and BCAA supplementation at amounts similar to our study did not affect pregnancy rates and litter size in rats [36] or mice [35], but, similar to our study, low-protein diets (<7% *w*/*w* wt/wt) reduced litter size and increased cannibalism in mice [55]. It is plausible that the supplementation of BCAAs under conditions of dietary protein restriction and high-fat feeding may induce metabolic stress and disrupt neuroendocrine balance. For example, elevated BCAA levels have been linked to activation of mTOR, causing insulin resistance [56] and disruption of neuronal signaling pathways that exert anxiety-like behavior in rats [57]. Further, neuroendocrine disturbances can elevate stress or suppress prolactin and oxytocin [58,59,60], lead to maladaptive behaviors such as cannibalism at low dietary protein concentrations in mice [55], and such stressors may further compound metabolic imbalances and lead to insufficient nutrient allocation to offspring under obesogenic high-fat diet-fed conditions.

Protein restriction during critical developmental periods, such as pregnancy and lactation, has been shown to adversely affect overall offspring growth. Offspring born to protein-restricted mothers consistently exhibit lower birth weights compared to those from mothers who are fed a normal protein diet, a finding that has been widely reported in the literature [16,61,62,63,64]. Our results align with these observations, as pups from protein-restricted dams had reduced birth weights. In addition to lower birth weight, the weight trajectory of protein-restricted offspring was markedly different from that of controls, persisting from birth through early adulthood. Previous studies have similarly reported pre- and post-weaning growth restrictions in pups subjected to maternal protein deficiency [8,65,66]. For instance, 8% maternal dietary protein resulted in a 58% reduction in weight gain in restricted pups [61], and 6% maternal dietary protein reduced fetal weights at gestational day 20 [62]. In contrast, others [63,64] used 8–9% protein in Wistar rats and did not observe differences in birth weight; however, they did report postnatal growth impairments, particularly from days 4 to 35 and days 21 to 84, respectively. However, none of these studies evaluated the effects of dietary protein restriction under obesogenic conditions. For the first time, we show that pups from high-fat-fed, protein-restricted mothers exhibited both lower birth weights and impaired growth for up to 8 weeks of age. However, in contrast to a previous study with normal diets in rats [36], but in agreement with other studies in mice [35] and a meta-analysis [67], BCAA supplementation was ineffective in reversing birth weight or postnatal growth deficits in offspring from protein-restricted dams in our study. Notably, given the rising prevalence of LBW in obese pregnancies [5,6,7], findings on LBW offspring from mothers fed low-protein, high-fat diets in our study provide clinical relevance to our model and a basis for investigating potential strategies to mitigate or reverse the effects of early-life protein deficiency under obesogenic conditions.

Beyond body weight and growth, bone development is significantly affected by protein restriction, further emphasizing the broad impact of early-life nutritional insults. We demonstrate that maternal protein restriction affected cranial size, resulting in reduced length and width, but BCAA supplementation was ineffective in rescuing such cranial deficits. Our findings are consistent with previous studies [45,61,68] showing that maternal protein restriction during lactation reduced skull height, length, and width in rat and mouse pups. These morphometric alterations suggest impaired development of the neurocranium, affecting brain growth, as well as the viscerocranium, which plays a critical role in respiration and feeding [45,61]. Together, our findings reveal that offspring of protein-restricted dams had low birth weight, reduced postnatal weight gain, and impaired neurocranial growth that persists into adulthood.

Our study has several caveats. The litter sizes were much smaller in our studies relative to previous reports [35,36,45]. Whether a high-fat and low-protein diet impairs conception and reproductive performance remains to be ascertained. The occurrence of cannibalism in the BCAA-supplemented group reduced the number of animals surviving to weaning. This also limited our ability to determine whether the observed craniofacial deformities were incidental or a direct consequence of maternal protein restriction.

## 5. Conclusions

Overall, our study demonstrates that maternal protein restriction, with or without BCAA supplementation, increases maternal energy expenditure and lipid utilization but impairs reproductive success and offspring growth and development. Although two different doses of BCAA resulted in some metabolic improvements in the dams, they were insufficient to reverse the adverse effects of maternal obesity on LBW, offspring viability, growth, and skeletal development. Future studies exploring alternative dosing strategies may help identify more effective approaches to mitigate these outcomes. In summary, our findings highlight the complex, albeit opposing, effects of protein restriction and BCAA supplementation on maternal adaptation and offspring development, emphasizing the critical role of maternal nutrition during pregnancy and lactation in shaping postnatal health outcomes.

## Figures and Tables

**Table 1:** Diet composition.

	Study 1			Study 2		
Ingredients	CON	LP	LP + 100% BCAA	CON	LP	LP + 200% BCAA
Corn oil	60	60	60	60	60	60
Lard	145	145	145	145	145	145
Casein	230	55	55	230	35	35
Corn starch	315.7	490.7	456.7	315.7	428.7	428.7
Sucrose	50	50	50	50	50	50
Fructose	100	100	100	100	100	100
α-Cellulose	50	50	50	50	50	50
AIN-93G-MX mineral mix	35	35	35	35	35	35
AIN-93-VX vitamin mix	10	10	10	10	10	10
L-Cysteine	1.8	1.8	1.8	1.8	1.8	1.8
Choline bitartrate	2.5	2.5	2.5	2.5	2.5	2.5
tert-Butylhydroquinone (TBHQ)	0.008	0.008	0.008	0.008	0.008	0.008
Leu			15.4			37.7
Ala					82.28	
Ile			8.3			20.33
Val			9.9			24.25
Total amount (g)	1000	1000	1000	1000	1000	1000
Casein AA profile						
Leu	20.24	4.84	4.84	20.24	3.08	3.08
Arg	8.28	1.98	1.98	8.28	1.26	1.26
Asp	14.95	3.575	3.575	14.95	2.275	2.275
Glu	47.84	11.44	11.44	47.84	7.28	7.28
His	5.98	1.43	1.43	5.98	0.91	0.91
Met	5.98	1.43	1.43	5.98	0.91	0.91
Phe	11.5	2.75	2.75	11.5	1.75	1.75
Thr	8.74	2.09	2.09	8.74	1.33	1.33
Trp	2.76	0.66	0.66	2.76	0.42	0.42
Ala	5.98	1.43	1.43	5.98	0.91	0.91
Cys	0.92	0.22	0.22	0.92	0.14	0.14
Gly	4.14	0.99	0.99	4.14	0.63	0.63
Ile	11.04	2.64	2.64	11.04	1.68	1.68
Lys	17.02	4.07	4.07	17.02	2.59	2.59
Pro	26.91	6.435	6.435	26.91	4.095	4.095
Ser	12.42	2.97	2.97	12.42	1.89	1.89
Tyr	12.19	2.915	2.915	12.19	1.855	1.855
Val	13.11	3.135	3.135	13.11	1.995	1.995
Composition						
Protein (% Kcal)	20%	5%	8%	20%	10%	10%
Carbohydrate (% Kcal)	40%	55%	52%	40%	50%	50%
Fat (% Kcal)	40%	40%	40%	40%	40%	40%
Total calories/g	4.63	4.63	4.63	4.63	4.63	4.63

**Table 2:** Maternal reproductive performance and offspring viability.

	Study 1		
	Control	LP	LP + 100%BCAA
Successful pregnancies (%)	50	18.18	27.27
Litter size	14.5	14.5	11 *
Pups born alive (%)	98.28	96.55	100
Pups born dead (%)	1.75	3.45	0
Pup survival until weaning (%)	94.83	96.55	54.55 ^b^
Cannibalism (mothers%)	0	0	33.33
	Study 2		
	Control	LP	LP + 200%BCAA
Successful pregnancies (%)	75	50	75
Litter size	13.66	7 ^a^	11.16 *
Pups born alive (%)	100	100	86.57 ^b^
Pups born dead (%)	0	0	13.43 ^b^
Pup survival until weaning (%)	89.02	90.48	0.0 ^b^
Cannibalism (mothers%)	16.67	0	83.33 *

* *p* < 0.1 control vs. BCAA; ^a^ *p* < 0.05 control vs. LP; ^b^ *p* < 0.05 control vs. BCAA; Study 1: one mother died during birth and one aborted.

## Data Availability

The data that support the findings of this study are available from the corresponding author, P.K. Chelikani, upon reasonable request.

## Supplementary Materials

The following supporting information can be downloaded at https://www.mdpi.com/article/10.3390/nu18020322/s1, Figure S1 (Effects of maternal dietary interventions in study 1 on energy expenditure and respiratory quotient on days 1–5 of gestation), Figure S2 (Effects of maternal dietary interventions in study 1 on energy expenditure and respiratory quotient on day −1 prior to parturition, and days 1 and 3 of lactation), Figure S3 (Effects of maternal dietary interventions in study 2 on energy expenditure and respiratory quotient on days 1–5 of gestation), Figure S4 (Effects of maternal dietary interventions in study 2 on energy expenditure and respiratory quotient on day −1 prior to parturition, and days 1 and 3 of lactation, Figure S5 (Effects of maternal dietary interventions on dam and representative offspring at 2 weeks of age in shoe box cages).

## Abbreviations

The following abbreviations are used in this manuscript: ANOVA—Analysis of Variance, BCAA—Branched-Chain Amino Acids, CLAMS—Comprehensive Laboratory Animal Monitoring System, CT—Computed Tomography, DIO—Diet-Induced Obese, Energy Expenditure, EE—Energy Expenditure, FL—Facial Length, FW—Facial Width, HFD—High-Fat Diet, LBW—Low Birth Weight, LP—Low-Protein diet, NCW—Neurocranial Width, NCH—Neurocranial Height, IACUC—Institutional Animal Care and Use Committee, IP—Intraperitoneal, IPGTT—Intraperitoneal Glucose Tolerance Test, IPITT—Intraperitoneal Insulin Tolerance Test, RQ—Respiratory Quotient, SD—Sprague Dawley, SEM—Standard Error of the Mean, SB—Skull Base, SL—Skull Length, SW—Skull Width, SPSS—Statistical Package for the Social Sciences, tBHQ—Tert-Butylhydroquinone, VO_2_—Volume of Oxygen Consumed, VCO_2_—Volume of Carbon Dioxide Produced.
