# Supplementary material for: Maternal Protein Restriction and Branched-Chain Amino Acid Supplementation Differentially Affect Maternal Energy Balance and Impair Offspring Growth
Source: Nutrients. 2026 Jan 20;18(2):322. doi: 10.3390/nu18020322 (PMC12844609; doi:10.3390/nu18020322)

SUPPLEMENTARY INFORMATION

Title: Maternal Protein Restriction and Branched Chain Amino Acid Supplementation Differentially Affect Maternal Energy Balance and Impair Offspring Growth.

Authors: Daniela Redrovan [1], Souvik Patra [1,#], Md Tareq Aziz [1], Matthew W. Gorton [2], Emily A. Chavez [1], Scott Frederiksen [1], Joshua Rowe [1], Adel Pezeshki [2] and Prasanth K. Chelikani [1,*]

Affiliation: [1]School of Veterinary Medicine, Texas Tech University, 7671 Evans Drive, Amarillo, Texas 79106, USA. [2]Department of Animal and Food Sciences, Oklahoma State University, Stillwater, OK 74078, USA. [#]Current: Van Andel Institute, Grand Rapids, MI 49503, USA.

Keywords: Protein restriction; Branched chain amino acids; Pregnancy and Lactation; Craniofacial growth; Low birth weight

Running title: Protein restriction and BCAA impact offspring growth.

***Corresponding Author:** Prasanth K. Chelikani, BVSc, MVSc, PhD, FTOS, School of Veterinary Medicine, Texas Tech University, 7671 Evans Drive, Amarillo, TX 79106, USA, E-mail: pchelika@ttu.edu, phone: +1 (806) 834-5697.

Funding: This work was supported by the American Heart Association (Grant# 953881), Diabetes Research and Action Education Foundation (Grant# 523), and the Texas Tech University School of Veterinary Medicine to Prasanth K. Chelikani.

Disclosure: The authors declare no conflicts of interest.

Supplemental Figure Legends

Figure S1. Effects of maternal dietary interventions in study 1 on: A-E) energy expenditure on days 1-5 of gestation, and F-J) respiratory quotient on days 1-5 of gestation. Pregnant diet-induced obese (DIO) SD rats were fed control HFD (n=8), low-protein (5% protein, n=11), and LP+BCAA (LP + 100% requirement for branched chain amino acids, n=11). $^{a,b}P < 0.05$ vs. control, $^{c}P < 0.05$ low-protein vs. LP + BCAA, $^{*}P < 0.10$ vs. control. Values are mean ± SEM, n = 8-11.

Figure S2. Effects of maternal dietary interventions in study 1 on: A-C) energy expenditure on day -1 prior to parturition, and days 1 and 3 of lactation, D-F) respiratory quotient on day -1 prior to parturition, and days 1 and 3 of lactation. Pregnant diet-induced obese (DIO) SD rats were fed control HFD (n=8), low-protein (5% protein, n=11), and LP+BCAA (LP + 100% requirement for branched chain amino acids, n=11). $^{a,b}P < 0.05$ vs. control, $^{c}P < 0.05$ low-protein vs. LP + BCAA, $^{*}P < 0.10$ vs. control. Values are mean ± SEM, n = 8-11.

Figure S3. Effects of maternal dietary interventions in study 2 on: A-E) energy expenditure on days 1-5 of gestation, and F-J) respiratory quotient on days 1-5 of gestation. Pregnant diet-induced obese (DIO) SD rats were fed control HFD (20% protein, n=8), low-protein (10% protein, LP; n=8), and LP+2BCAA (LP+200% requirement for of branched chain amino acids; n=8) during pregnancy and lactation. $^{a,b}P < 0.05$ vs. control, $^{c}P < 0.05$ low-protein vs. LP + BCAA, $^{*}P < 0.10$ vs. control. Values are mean ± SEM, n = 8.

Figure S4. Effects of maternal dietary interventions in study 2 on: A-C) energy expenditure on day -1 prior to parturition, and days 1 and 3 of lactation, D-F) respiratory quotient on day -1 prior to parturition, and days 1 and 3 of lactation. Pregnant diet-induced obese (DIO) SD rats were fed control HFD (20% protein, n=8), low-protein (10% protein, LP; n=8), and LP+2BCAA (LP+200% requirement for of branched chain amino acids; n=8) during pregnancy and lactation. $^{a,b}P < 0.05$ vs. control, $^{*}P < 0.10$ vs. control. Values are mean ± SEM, n = 8.

Figure S5. Effects of maternal dietary interventions on representative offspring at 2 weeks of age in shoe box cages. A) Control dam with her litter, and B) Low protein litter.

Supplementary Information: *Redrovan et al. Maternal Protein Restriction and Branched Chain Amino Acid Supplementation Differentially Affect Maternal Energy Balance and Impair Offspring Growth.*

Figure S1.

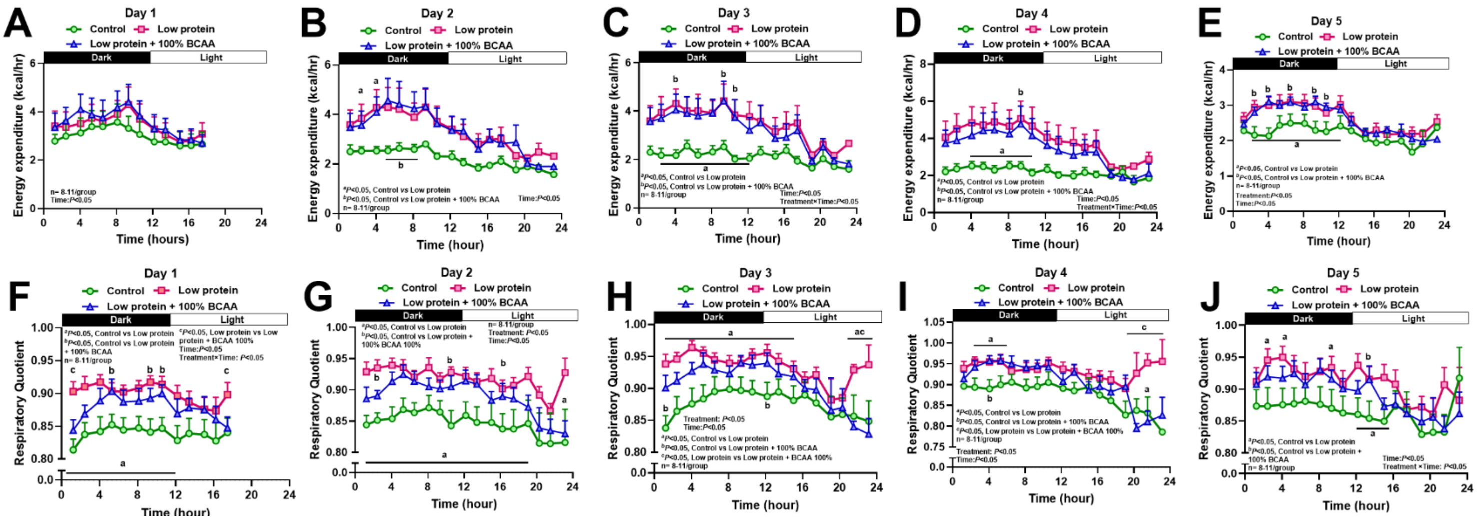

Figure S2.

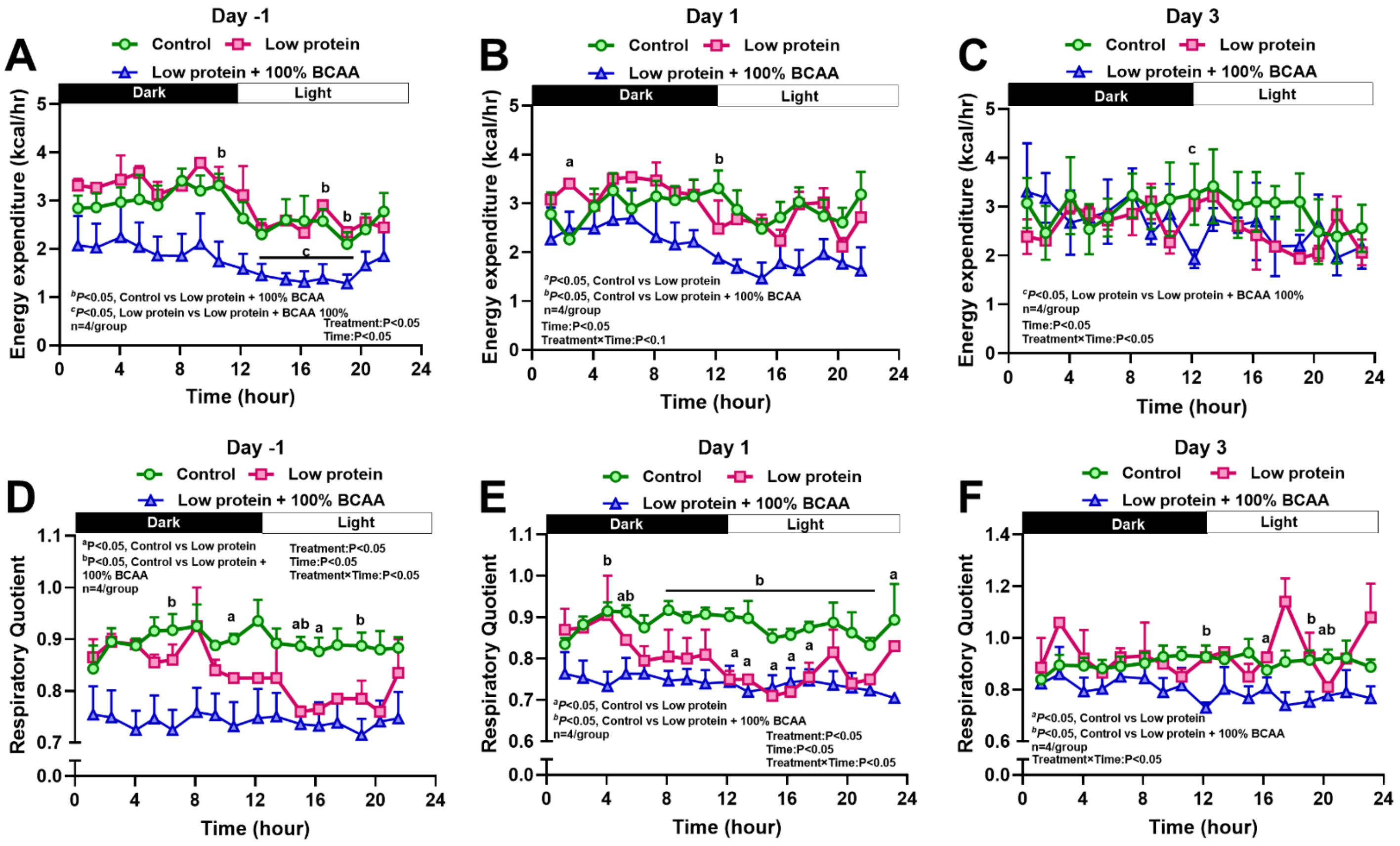
A
Day -1
Control
Low protein
Low protein + 100% BCAA
Dark
Light
Energy expenditure (kcal/hr)
Time (hour)
bP<0.05, Control vs Low protein + 100% BCAA
cP<0.05, Low protein vs Low protein + BCAA 100%
n=4/group
Treatment:P<0.05
Time:P<0.05
B
Day 1
aP<0.05, Control vs Low protein
bP<0.05, Control vs Low protein + 100% BCAA
n=4/group
Time:P<0.05
Treatment×Time:P<0.1
C
Day 3
cP<0.05, Low protein vs Low protein + BCAA 100%
n=4/group
Time:P<0.05
Treatment×Time:P<0.05
D
Day -1
Respiratory Quotient
aP<0.05, Control vs Low protein
bP<0.05, Control vs Low protein + 100% BCAA
n=4/group
Treatment:P<0.05
Time:P<0.05
Treatment×Time:P<0.05
E
Day 1
aP<0.05, Control vs Low protein
bP<0.05, Control vs Low protein + 100% BCAA
n=4/group
Treatment:P<0.05
Time:P<0.05
Treatment×Time:P<0.05
F
Day 3
aP<0.05, Control vs Low protein
bP<0.05, Control vs Low protein + 100% BCAA
n=4/group
Treatment×Time:P<0.05

Supplementary Information: *Redrovan et al. Maternal Protein Restriction and Branched Chain Amino Acid Supplementation Differentially Affect Maternal Energy Balance and Impair Offspring Growth.*

Figure S3.

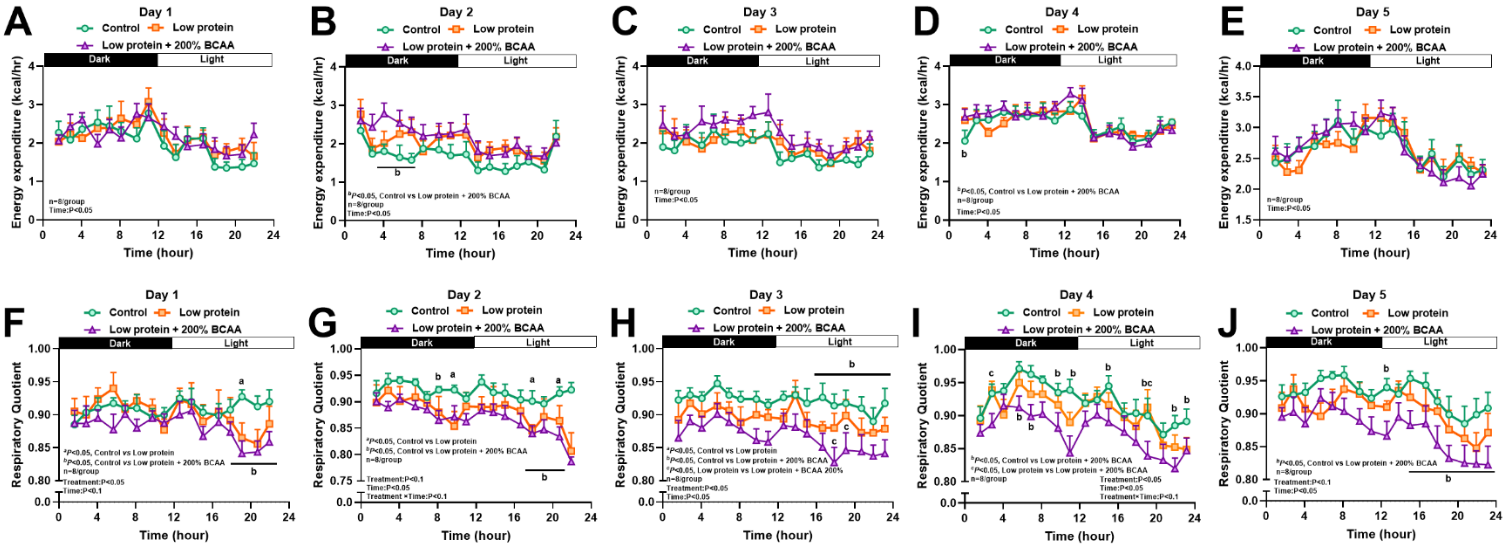

Supplementary Information: *Redrovan et al. Maternal Protein Restriction and Branched Chain Amino Acid Supplementation Differentially Affect Maternal Energy Balance and Impair Offspring Growth.*

Figure S4.

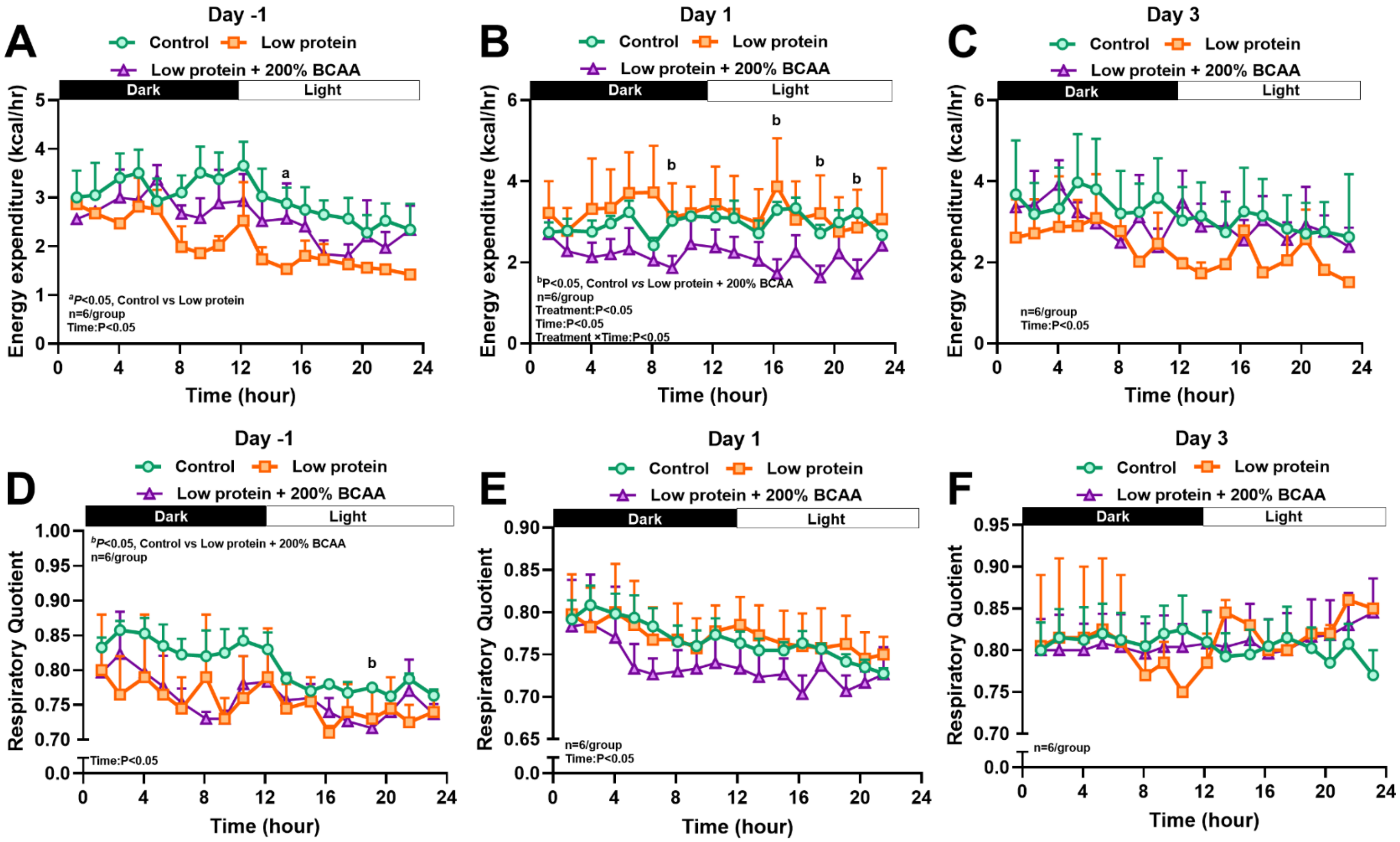

Figure S5.

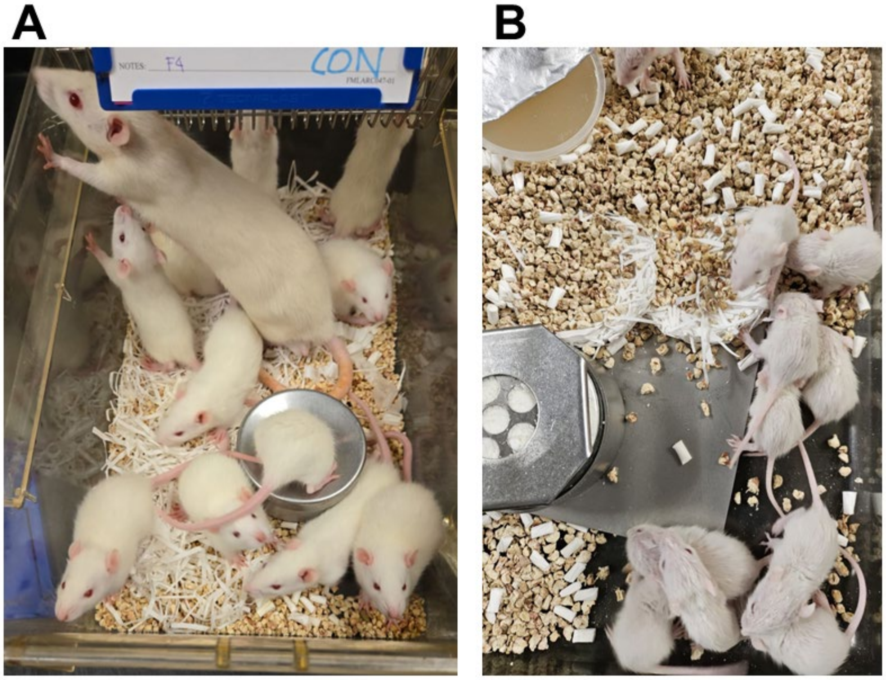

Supplement: Supplementary file 1 [file nutrients-18-00322-s001.zip › nutrients-4026637-supplementary.pdf]
